# Supplementary material for: Multi-proxy evidence for sea level fall at the onset of the Eocene-Oligocene transition
Source: Nat Commun. 2023 Aug 8;14:4748. doi: 10.1038/s41467-023-39806-6 (PMC10409788; doi:10.1038/s41467-023-39806-6)

Supplementary Information for

Multi-proxy evidence for sea level fall at the onset of the Eocene-Oligocene transition

Marcelo A. De Lira Mota, Tom Dunkley Jones, Nursufiah Sulaiman, Kirsty M. Edgar, Tatsuhiko Yamaguchi, Melanie J. Leng, Markus Adloff, Sarah E. Greene, Richard Norris, Bridget Warren, Grace Duffy, Jennifer Farrant, Masafumi Murayama, Jonathan Hall, James Bendle

Correspondence to: marcelomota@usp.br

This file includes:

Figures S1 to S3 and captions

Figure S1. Multi-proxy records from the Mossy Grove Core (MGC) in depth and age domains: Total organic carbon, carbonate fine-fraction (<20 µm) $\delta^{13}C$ and $\delta^{18}O$ records, and selected indicator of continental influence – sporomorph-to-dinocyst ratio. Key: epoch – Pleistocene (Pl.); lithostratigraphic units – Forest Hill (FH), Moodys Branch Formation (MB), Cockfield Formation (Cf).

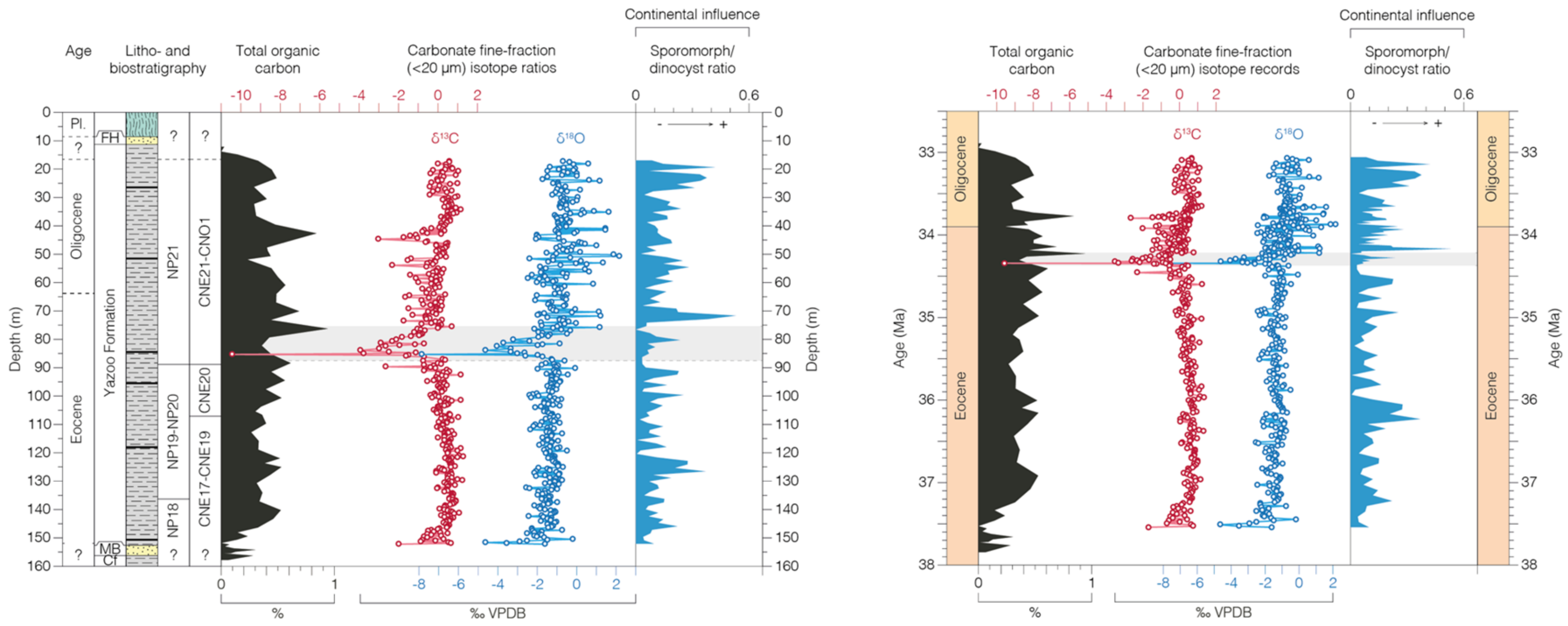

Figure S2. Microscopic images of *Cibicidoides* sp. (1, 2, 5) and *Uvigerina jacksonensis* Cushman (3, 4, 6) from sample MGC98 (29.9 m: 33.52 Ma): 1-3: scanning electron microscope (SEM) image of individual specimens; 4: SEM image of the specimen with cut off apical end; 5-6: optical microscopic image under reflected light. Scale bars: 100 µm.

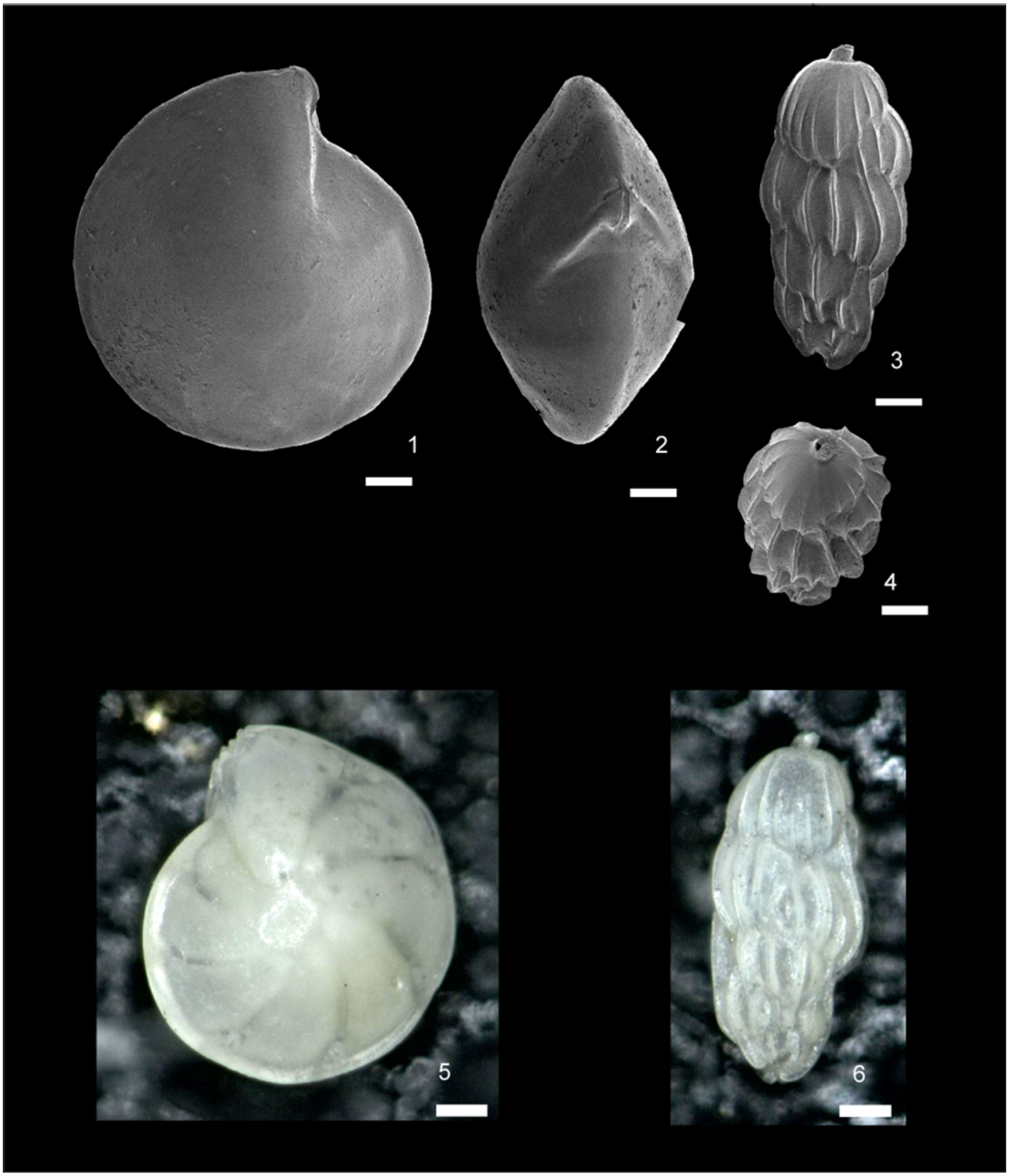

Figure S3. Simplified $\delta^{13}C$ curve across the negative isotope excursion (NIE; top), the respiration rates of organic carbon required by cGENIE to produce this NIE in marine dissolved inorganic carbon (DIC; middle), and global mean carbonate saturation horizon (CSH) depth changes simulated in response to the carbon respiration (bottom).

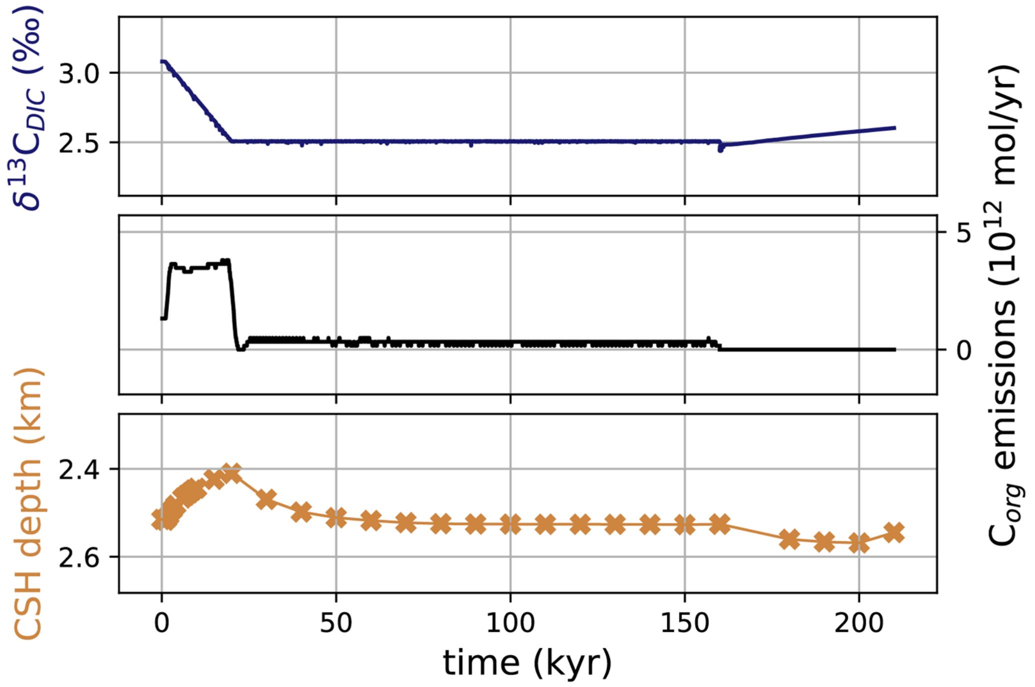

Supplement: Supplementary file 1 — Supplementary Information [file 41467_2023_39806_MOESM1_ESM.pdf]
